# Supplementary material for: A Bibliometric Analysis of Stroke Caregiver Research from 1989 to 2022
Source: Int J Environ Res Public Health. 2023 Mar 6;20(5):4642. doi: 10.3390/ijerph20054642 (PMC10001807; doi:10.3390/ijerph20054642)
Supplement: Supplementary file 1 [file ijerph-20-04642-s001.zip › ijerph-2208557-supplementary.pdf]

Supplementary Materials

1. Countries

Supplement Table S1: Number of Publication by Countries and Income Group

Rank (No. Publication)	Country	No. of Publication	Total Citation	Average Citation	World Bank Income Group 2019
1	USA	193	4720	24.46	High Income
2	China	82	1240	15.12	Upper Middle Income
3	Canada	41	1455	35.49	High Income
4	Netherlands	33	1183	35.85	High Income
5	UK	29	1472	50.76	High Income
6	Brazil	24	177	7.38	Upper Middle Income
7	Australia	23	590	25.65	High Income
8	Turkey	21	131	6.24	Upper Middle Income
9	Korea	18	319	17.72	High Income
= 9	Singapore	18	220	12.22	High Income
11	Iran	15	112	7.47	Upper Middle Income
= 11	Italy	15	289	19.27	High Income
= 11	Sweden	15	737	49.13	High Income
14	Germany	13	112	8.62	High Income
= 14	India	13	160	12.31	Lower Middle Income
16	Spain	12	130	10.83	High Income
17	Nigeria	11	153	13.91	Lower Middle Income
18	Malaysia	10	15	1.5	Upper Middle Income
19	Portugal	9	77	8.56	High Income
= 19	South Africa	9	28	3.11	Upper Middle Income
21	Japan	8	214	26.75	High Income
22	Thailand	7	65	9.29	Upper Middle Income
23	Indonesia	6	9	1.5	Lower Middle Income
24	Luxembourg	5	91	18.2	High Income
25	Belgium	4	84	21	High Income
= 25	Poland	4	132	33	High Income
27	Greece	3	7	2.33	High Income
= 27	Ireland	3	46	15.33	High Income
= 27	Israel	3	44	14.67	High Income
= 27	Pakistan	3	10	3.33	Lower Middle Income
31	France	2	11	5.5	High Income
= 31	New Zealand	2	40	20	High Income
= 31	Norway	2	7	3.5	High Income
= 31	Uganda	2	11	5.5	Low Income
35	Argentina	1	1	1	Upper Middle Income
= 35	Austria	1	0	0	High Income
= 35	Benin	1	10	10	Lower Middle Income
= 35	Cameroon	1	0	0	Lower Middle Income
= 35	Congo	1	0	0	Lower Middle Income
= 35	Denmark	1	277	277	High Income
= 35	Finland	1	149	149	High Income
= 35	Ghana	1	3	3	Lower Middle Income
= 35	Iceland	1	4	4	High Income
= 35	Jamaica	1	6	6	Upper Middle Income
= 35	Jordan	1	19	19	Upper Middle Income

Rank (No. Publication)	Country	No. of Publication	Total Citation	Average Citation	World Bank Income Group 2019
= 35	Latvia	1	0	0	High Income
= 35	Lebanon	1	2	2	Upper Middle Income
= 35	Saudi Arabia	1	8	8	High Income
= 35	Switzerland	1	3	3	High Income
= 35	Vietnam	1	7	7	Lower Middle Income
= 35	Zimbabwe	1	2	2	Lower Middle Income

Income Group Source (2019):

- Source Page: https://datahelpdesk.worldbank.org/knowledgebase/articles/906519
- Excel File: http://databank.worldbank.org/data/download/site-content/OGHIST.xlsx

2. Institution Collaboration

Supplement Table S2: Institution Collaboration Cluster by Colour (figure 7)

**cluster 1 (colour: red)**
univ calgary
univ toronto
mcmaster univ
univ washington
michigan state univ
univ delaware
indiana univ
indiana univ purdue univ
univ cincinnati
no arizona univ
univ n carolina
boston coll
univ roma tor vergata
univ florida
schmitzberger
geriatric res educ and clin ctr
univ miami
res serv
penn state univ

**cluster 2 (colour: dark blue)**
baylor coll med
univ texas hlth sci ctr houston
texas woman univ
emory univ
univ alabama
univ alabama birmingham
univ s florida
johns hopkins univ

**cluster 3 (colour: orange)**
univ illinois
rehabil inst chicago
northwestern univ
yonsei univ
vrije univ amsterdam
inst rehabil res
maastricht univ
univ groningen
univ med ctr utrecht
leiden univ
univ appl sci
charite univ med berlin
univ hosp wurzburg

**cluster 4 (colour: purple)**
duke univ
china med univ
univ oxford
duke kunshan univ
ningxia med univ
peking univ
chongqing med univ
george inst global hlth
univ sydney
singapor inst technol
chinese univ hong kong

**cluster 5 (colour: light blue)**
gleaneagles hosp
nanyang technol univ
durham vet adm med ctr
chou neurosci clin
off healthcare transformat
technol and data grp

**cluster 6 (colour: green)**
singapore gen hosp
changi gen hosp
natl neurosci inst
natl univ singapore hosp
raffles neurosci c tr
khoo teck puat hosp
durham va med ctr
st andrews community hosp
tan tock seng hosp
natl univ hosp
duke nus grad med school
st lukes hosp
mt alvernia hosp
policy res and econ off
lawson htlh res inst

**cluster 7 (colour: grey)**
chang gung univ
sch nursing
asia univ

**cluster 8 (colour: pink)**
lund univ
karolinska inst
uppsala univ
clin res ctr
univ sains malaysia
king coll london
univ leeds
acad unit elderly care and rehabil

**cluster 9 (colour: light brown)**
vrije univ brussel
univ antwerp
univ melbourne
monash univ

**cluster 10 (colour: dark brown)**
hong kong polytech univ
zhenzou univ

**cluster 11 (colour: light green)**
tianjin med univ
sichuan univ

3. R script

We believe in research reproducibility, and to honour that belief, R script used in this manuscript is shared here. Reader may run this script to validate our findings.

Data available from https://zenodo.org/record/7421945

```
## Library
require(tidyverse)
require(bibliometrix)

## Data Import and Convert------
## File link from zenodo site
linkfile_1 <- "https://zenodo.org/record/7421945/files/22-12-07%20WOS_TIstcgp1.bib?download=1"
linkfile_2 <- "https://zenodo.org/record/7421945/files/22-12-07%20WOS_TIstcgp2.bib?download=1"
## Import and Combine
bibds1_wos <- convert2df(file = linkfile_1, dbsource = "wos", format = "bibtex")
bibds2_wos <- convert2df(file = linkfile_2, dbsource = "wos", format = "bibtex")
bibds_wos <- bind_rows(bibds1_wos, bibds2_wos)
## Convert to 'bibliometrix' class object
bibres <- biblioAnalysis(bibds_wos)

## General Information ------
## Summary
bibres_summary <- summary(bibres)
## Summary Plot
bibres_plot <- plot(bibres, k = 10)
## Figure 1
bibres_plot$AnnualScientProd

## Influential Articles -----
## Table 1
tibble(Title = bibds_wos$TI, Author = bibds_wos$AU, Year = bibds_wos$PY,
       DOI = bibds_wos$DI, Citations = bibds_wos$TC,
       CitaPerYear = Citations/(2023-Year)) %>%
  mutate(across(.cols = c(Title, Author), .fns = str_to_title),
         CitaPerYear = round(CitaPerYear, 2)) %>%
  arrange(desc(Citations)) %>% head(n=10)
## Table 2
tibble(Title = bibds_wos$TI, Author = bibds_wos$AU, Year = bibds_wos$PY,
       DOI = bibds_wos$DI, Citations = bibds_wos$TC,
       CitaPerYear = Citations/(2023-Year)) %>%
  mutate(across(.cols = c(Title, Author), .fns = str_to_title),
         CitaPerYear = round(CitaPerYear, 2)) %>%
  arrange(desc(CitaPerYear)) %>% head(n=10)

## Language -----
## Table 3
bibds_wos %>%
  group_by(LA) %>% summarise(n = n()) %>%
```

```
  mutate(percent = n / sum(n) * 100, percent = round(percent,1)) %>%
  arrange(desc(n))

## Authors -----
## Figure 2
bibds_noaufreq <- bibds_wos %>%
  select(TI, AU, DT) %>% tibble() %>%
  mutate(no_auth = str_count(AU, pattern = ";") + 1,
         DT = fct_recode(DT,
                         "ARTICLE" = "PROCEEDINGS PAPER",
                         "ARTICLE" = "ARTICLE; PROCEEDINGS PAPER",
                         "ARTICLE" = "ARTICLE; EARLY ACCESS",
                         "REVIEW" = "REVIEW; EARLY ACCESS")) %>%
  rename("paper" = "TI", "author" = "AU", "type" = "DT") %>%
  group_by(no_auth, type) %>%
  summarise(freq = n(), .groups = "drop") %>%
  mutate(percent = freq / sum(freq) * 100, percent = round(percent,1))
bibds_noaufreq %>%
  mutate(type = str_to_title(type)) %>%
  ggplot(aes(no_auth, freq, fill = type)) +
  geom_bar(stat = "identity") +
  labs(x = "Number of Authors", y = "Frequency (Number of Articles)",
  fill = "Type") +
  scale_x_continuous(breaks = seq(0,30,4)) +
  scale_y_continuous(breaks = seq(0,200,20)) +
  theme_bw() + theme(legend.position = "top")
## Table 4
bibres_aulist <- bibres$Authors
bibres_autable <- tibble(Rank = seq_along(bibres_aulist),
                         Author = rownames(bibres_aulist),
                         Np = as.integer(bibres_aulist)) %>%
  mutate(Author = fct_reorder(Author, Rank))
bibres_autable %>%
  rename("Number of Publications" = "Np") %>%
  mutate(Author = stringr::str_to_title(Author),
         percent = `Number of Publications` / bibres$Articles * 100,
         percent = round(percent,1)) %>% head(n = 15)
## Figure 3
bib_AuCoupling_NetMatrix <- biblioNetwork(bibds_wos, analysis = "coupling",
                                          network = "authors", sep = ";")
bib_AuCoupling_Plot <- networkPlot(bib_AuCoupling_NetMatrix, n = 25,
                                   cluster = "optimal", type = "auto",
                                   size.cex = T, size = 20, remove.multiple = F,
                                   Title = "Bibliographic coupling of the authors",
                                   alpha = .7)
```

```
## Keywords -----
## Table 5
cbind(Rank = 1:10, bibres_summary$MostRelKeywords)
## Figure 4
bib_kwco_NetMatrix <- biblioNetwork(bibds_wos, analysis = "co-occurrences",
                                    network = "keywords", sep = ";")
bib_kwco_Plot <- networkPlot(bib_kwco_NetMatrix, normalize = "association",
                             n = 25, Title = "Keyword Co-occurences",
                             cluster = "optimal", type = "fruchterman",
                             size.cex = T, size = 15,  remove.multiple = F,
                             edgesize = 7, labelsize = 1.2, label.cex = F,
                             label.n = 20, edges.min = 10)

## Journals -----
## Table 6
bib_bradford <- bradford(bibds_wos)
bib_bradfordtable <- bib_bradford$table %>%
  select(Zone, Freq, Rank) %>% tibble() %>% group_by(Zone) %>%
  summarise(nSO = n(), nArt = sum(Freq),
            RankRange = str_c(min(Rank), max(Rank), sep = "-")) %>%
  mutate(percent = nArt / sum(nArt) * 100, percent = round(percent,1))
bib_bradford$table %>%
  filter(Zone == "Zone 1") %>% tibble() %>%
  mutate(SO = stringr::str_to_title(SO)) %>%
  rename("Journal Name" = "SO") %>% relocate(Rank)
## Figure 5
bib_CRSO <- metaTagExtraction(bibds_wos, Field = "CR_SO", sep = ";")
bib_CRSO_NetMatrix <- biblioNetwork(bib_CRSO, analysis = "co-citation",
                                    network = "sources", sep = ";")
bib_CRSO_Plot <- networkPlot(bib_CRSO_NetMatrix, n = 25,
                             Title = "Co-citation Network", type = "auto",
                             size.cex = T, size = 20, remove.multiple = F,
                             labelsize = 1, edgesize = 5, edges.min = 1, alpha = .7)

## Institutions -----
## Table 7
bibres_instlist <- bibres$Affiliations
bibres_insttable <- tibble(Rank = seq_along(bibres_instlist),
                           InstitutionAffiliation = rownames(bibres_instlist),
                           Np = as.integer(bibres_instlist)) %>%
  mutate(InstitutionAffiliation = fct_reorder(InstitutionAffiliation, Rank))
bibres_insttable %>%
  rename("Number of Publications" = "Np",
         "Instituion Name" = "InstitutionAffiliation") %>%
  mutate(`Instituion Name` = stringr::str_to_title(`Instituion Name`)) %>%
  head(n = 10)
```

```
## Figure 6
bib_educolab_NetMatrix <- biblioNetwork(bibds_wos, analysis = "collaboration",
                                        network = "universities", sep = ";")
bib_educolab_Plot <- networkPlot(bib_educolab_NetMatrix, n = 100,
                                 cluster = "optimal", type = "auto",
                                 size.cex = F, size = 5, remove.multiple = F,
                                 labelsize=1, alpha = 1, edgesize = 2,
                                 edges.min = 1, remove.isolates = T,
                                 community.repulsion = 0, label = F,
                                 Title = "Institutions collaboration")

## Country -----
## Table 8a
bibres_countrylist <- bibres$Countries
bibres_countrytable <- tibble(Rank = seq_along(bibres_countrylist),
                              Country = rownames(bibres_countrylist),
                              Np = as.integer(bibres_countrylist)) %>%
  mutate(Country = fct_reorder(Country, Rank),
         percent = Np / sum(Np) * 100, percent = round(percent,1))
## Table 8b
bibressum_countrytable <- tibble(Rank = 1:10,
                                 bibres_summary$TCperCountries) %>%
  rename("Country" = "Country     ") %>%
  mutate(Country = str_trim(Country),
         Country = fct_reorder(Country, Rank),
         `Total Citations` = as.integer(`Total Citations`),
         `Average Article Citations` = as.double(`Average Article Citations`),
         percent = `Total Citations` / sum(`Total Citations`) * 100,
         percent = round(percent,1)) %>%
  inner_join(x = ., y = select(bibres_countrytable, Country, Np), by = "Country") %>%
  relocate(percent, .after = `Total Citations`)
## Figure 7
bib_concolab <- metaTagExtraction(bibds_wos, Field = "AU_CO", sep = ";")
bib_concolab_NetMatrix <- biblioNetwork(bib_concolab, analysis = "collaboration",
                                        network = "countries", sep = ";")
bib_concolab_Plot <- networkPlot(bib_concolab_NetMatrix,
                                 n = dim(bib_concolab_NetMatrix)[1],
                                 type = "auto", Title = "Country Collaboration",
                                 size=10, size.cex=T, edgesize = 2, labelsize=1.1,
                                 edges.min = 2, remove.isolates = T,
                                 community.repulsion = 0, cluster = "none")
```
